# Evolution of Natural Lifespan Variation and Molecular Strategies of Extended Lifespan

**DOI:** 10.1093/geroni/igab046.119

**Published:** 2021-12-17

**Authors:** Alaattin Kaya

**Affiliations:** Virginia Commonwealth University, Richmond, Virginia, United States

## Abstract

To understand the genetic basis and the selective forces acting on longevity, it is useful to employ ecologically diverse individuals of the same species, widely different in lifespan. This way, we may capture the experiment of Nature that modifies the genotype arriving at different lifespans. Here, we analyzed 76 ecologically diverse wild yeast isolates and discovered wide diversity of lifespan. We sequenced the genomes of these organisms and analyzed how their replicative lifespan is shaped by nutrients and transcriptional and metabolite patterns. By identifying genes, proteins and metabolites that correlate with longevity across these isolates, we found that long-lived strains elevate intermediary metabolites, differentially regulate genes involved in NAD metabolism and adjust control of epigenetic landscape through conserved, rare histone modifier. Our data further offer insights into the evolution and mechanisms by which caloric restriction regulates lifespan by modulating the availability of nutrients without decreasing fitness.

